# Functional expression of a single-chain antibody to ErbB-2 in plants and cell-free systems

**DOI:** 10.1186/1479-5876-4-39

**Published:** 2006-09-29

**Authors:** Patrizia Galeffi, Alessio Lombardi, Immacolata Pietraforte, Flavia Novelli, Monica Di Donato, Maria Sperandei, Andrea Tornambé, Rocco Fraioli, Aline Martayan, Pier Giorgio Natali, Maria Benevolo, Marcella Mottolese, Francisco Ylera, Cristina Cantale, Patrizio Giacomini

**Affiliations:** 1 ENEA BIOTEC-GEN, CR Casaccia Via Anguillarese 301, 00060 Rome, Italy; 2 Laboratory of Immunology, Regina Elena Cancer Institute CRS, Via delle Messi d'Oro 156, 00158 Rome, Italy; 3 Laboratory of Pathology, Regina Elena Cancer Institute, Istituti Fisioterapici Ospitalieri, Via E. Chianesi 53, 00144 Rome, Italy; 4 Roche Diagnostics GmbH, Nonnenwald 2, D-82372 Penzberg, Germany; 5 Department of Cell Biology and Neurosciences, Istituto Superiore di Sanità, Rome, Italy

## Abstract

**Background:**

Aberrant signaling by ErbB-2 (HER 2, Neu), a member of the human Epidermal Growth Factor (EGF) receptor family, is associated with an aggressive clinical behaviour of carcinomas, particularly breast tumors. Antibodies targeting the ErbB-2 pathway are a preferred therapeutic option for patients with advanced breast cancer, but a worldwide deficit in the manufacturing capacities of mammalian cell bioreactors is foreseen.

**Methods:**

Herein, we describe a multi-platform approach for the production of recombinant Single chain Fragments of antibody variable regions (ScFvs) to ErbB-2 that involves their functional expression in (a) bacteria, (b) transient as well as stable transgenic tobacco plants, and (c) a newly developed cell-free transcription-translation system.

**Results:**

An ScFv (ScFv800E6) was selected by cloning immunoglobulin sequences from murine hybridomas, and was expressed and fully functional in all the expression platforms, thereby representing the first ScFv to ErbB-2 produced in hosts other than bacteria and yeast. ScFv800E6 was optimized with respect to redox synthesis conditions. Different tags were introduced flanking the ScFv800E6 backbone, with and without spacer arms, including a novel Strep II tag that outperforms conventional streptavidin-based detection systems. ScFv800E6 was resistant to standard chemical radiolabeling procedures (i.e. Chloramine T), displayed a binding ability extremely similar to that of the parental monovalent Fab' fragment, as well as a flow cytometry performance and an equilibrium binding affinity (Ka approximately 2 × 10^8 ^M^-1^) only slightly lower than those of the parental bivalent antibody, suggesting that its binding site is conserved as compared to that of the parental antibody molecule. ScFv800E6 was found to be compatible with routine reagents for immunohistochemical staining.

**Conclusion:**

ScFv800E6 is a useful reagent for *in vitro *biochemical and immunodiagnostic applications in oncology, and a candidate for future *in vivo *studies.

## Background

The ErbB family of receptor tyrosine kinases includes four members (ErbB-1 or HER-1 through ErbB-4 or HER-4) that signal upon engagement in combinatorial dimeric receptor combinations. ErbB-2 is the shared (and preferred) heterodimerization partner, and acts as the master coordinator and integrator of signaling amplification. Aberrant ErbB signaling is causally involved in the pathogenesis of human tumors including astrocytomas, head and neck squamous cell carcinomas, breast, ovary and prostate cancers. ErbB-2 overexpression, most often caused by gene amplification, can be detected by immunohistochemistry in approximately 30% of breast carcinomas, is associated with an aggressive clinical course, and predictive of a worse prognosis [1].

Antibodies to the ectodomain of the receptor molecule have provided, among other approaches, a successful strategy to target the ErbB-2 pathway [1]. A recombinant, humanized antibody known as Trastuzumab-Herceptin^® ^was developed (reviewed in [1]), and is now regularly included, alone and in combination with chemotherapy, among the preferred therapeutic options for patients with advanced breast cancer.

Although whole antibody expression in mammalian cells results in the reliable production of industrial grade recombinant products, it has at least three drawbacks: safety (zoonotic diseases and oncogenic DNA sequences might be inadvertently shuttled through recombinant antibody preparations), size of the therapeutic molecule (preventing tissue penetration), and cost (mammalian cultures are very expensive bioreactors) [2,3]. In addition, concerns have been raised that the foreseen expansion in biopharmaceutical production will soon result in a worldwide deficit in manufacturing capacity [2]. Alternative production systems need to be actively explored.

Recombinant antibody fragments of small size, such as minibodies, diabodies, and single chain fragments of variable antibody regions (ScFvs) have been expressed in bacteria, yeast, plants [3-5] and, more recently, in prokaryotic cell-free expression systems [6-9]. Although these expression platforms effectively address the problem of size reduction, sequence reshuffling, *in vitro *synthesis, and folding in non-physiological conditions may hamper the binding efficiency of some recombinant antibodies. In addition, the risk of endotoxin carryover and adverse reactions to allergenic contaminants cannot be formally excluded, particularly with recombinant protein preparations from prokaryotic systems.

Limitations notwithstanding, plants most effectively address the problems of safety and cost, and are particularly suited to process scale-up [2,3]. Unfortunately, because 'molecular farming' is a very challenging task, only a limited number of phytoantibodies have been obtained so far, and only a few bear oncological interest ([10-12] and reviewed in [3]). To our knowledge, the only available ScFv to ErbB-2 engineered for plant expression is ScFv800E6, preliminarily characterized by us [13,14], whereas other recombinant antibody fragments to ErbB-2 have been exclusively expressed in bacteria or yeast [15-17].

Cell-free expression systems hold great promise for postgenomic applications [7,9]. Recent refinements make it also possible to produce bioactive, multiple disulfide-bonded proteins [18], including recombinant antibodies [6,9]. Their major limitation is the low yield reported by some authors in early studies [6,8].

In summary, because there is no optimal expression platform for the development and pharmacological use of recombinant proteins, and there are no preset rules for predicting whether or not a cloned immunoglobulin fragment will be functionally expressed, an ideal approach to the development of pharmaceutical-grade antibody fragments must integrate the best of the available technologies, and each reagent has to be developed keeping in mind versatility as the ultimate goal. In this report, we describe our approach to the generation of a new series of ScFv800E6 derivatives in expression systems alternative to mammalian cell culture. We have extensively characterized these reagents to demonstrate that (quite unusually) their binding efficiency is substantially unaffected by the introduction of epitope tags and expression in bacteria, plants, or a novel high-yield cell-free transcription-translation system that ensures disulfide link formation. It is argued that versatility is a key feature that should be actively selected, if recombinant antibodies are to be used for biotechnological applications.

## Methods

### Cell lines and antibodies

The murine monoclonal antibodies (mAb) W6/800E6 (in short, 800E6) and mAb 100A4, an IgG1 and IgG2a respectively, bind two distinct polypeptide epitopes in the extracellular portion of ErbB-2 [19]. They were used in all flow cytometry experiments at optimal pre-determined dilutions. Hybridoma 800E6 was used to clone Ig sequences. MAb 100A4 was used as a control in some experiments. The mAbs W6/32 and Ep3 [20] recognize class I Major Histocompatibility antigens and a melanoma antigen, respectively, and were also used as controls. ErbB-2 transfectants and neoplastic cell lines (Table 1) were previously characterized by others [21] and ourselves [19] for ErbB-2 expression. Monovalent Fab' fragments were prepared by papain (Sigma-Aldrich St. Louis, MO, USA) digestion.

### Construction and features of recombinant ScFvs

**Figure 1:**
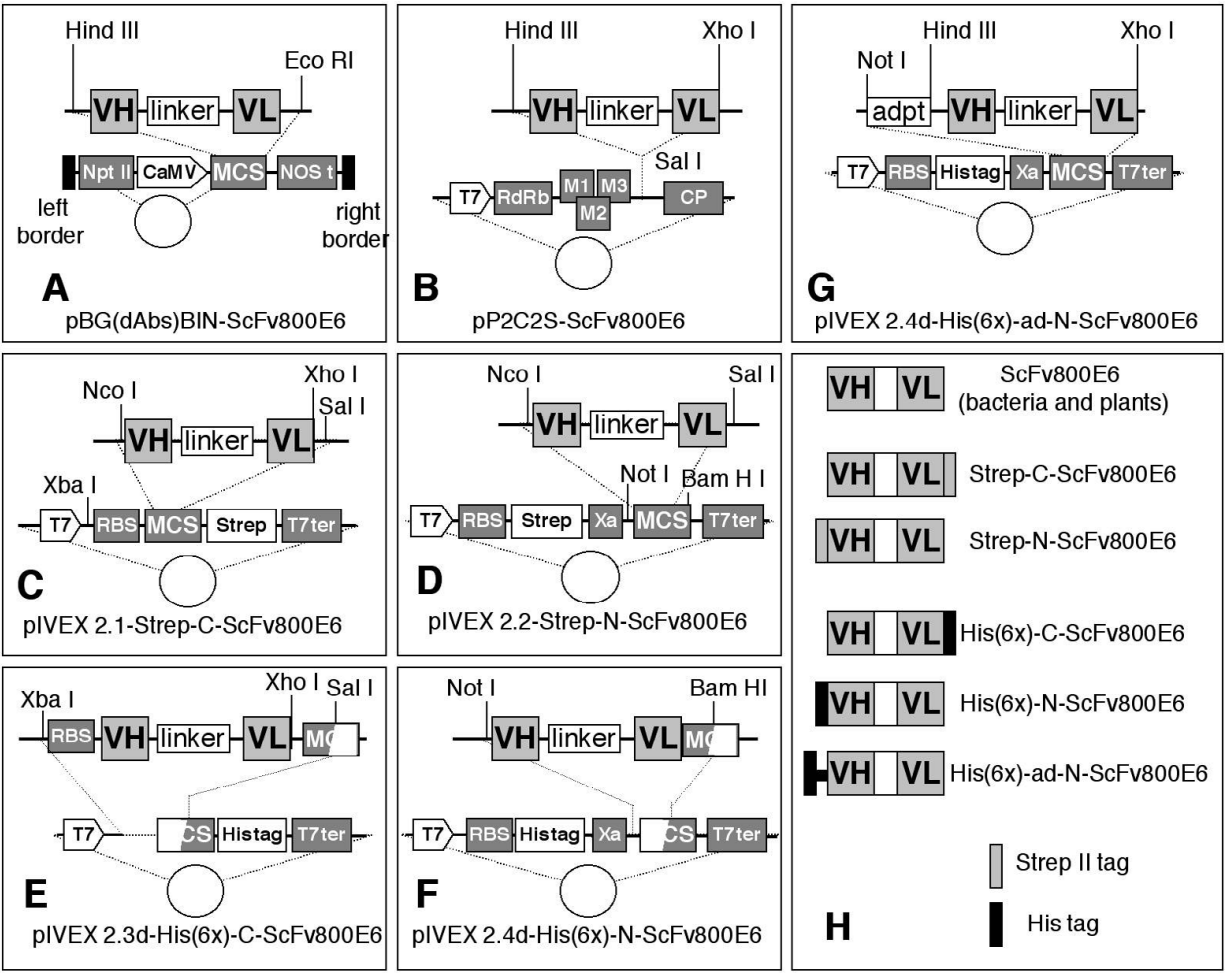
**Cloning strategy and map of ScFv 800E6 constructs**. Diagram illustrating the construction of ScFvs for expression in plants (stable and transient expression: panels A and B, respectively), and in cell-free transcription-translation systems (C–G). A linker peptide sequence (Gly_4_Ser)_3_, multiple cloning sites (MCS), the T7 promoter, the IPTG-inducible pLac promoter, a 3' transcription terminator, a proteolytic cleavage site (Xa), a ribosome binding site (RBS), the Strep II and His-tags, the kanamycin resistance NPT II gene, the 35S-CaMV promoter, the NOS terminator region for transcript stabilization, the RNA-dependent RNA-polymerase binding site (RdRb), the viral movement proteins (M1, M2 and M3), and the viral coat protein (CP) are present in different plasmids. A synopsis of the different ScFvs and tag positions is provided in panel H.

The cloning of Variable Light (VL) and Variable Heavy (VH) chain Ig sequences from the 800E6 hybridoma into the pEMBL-ScFv800E6 and pHEN (for prokaryotic expression) vectors has been described [13]. The pEMBL-ScFv800E6 plasmid was used to generate all the remaining constructs, depicted in figure 1. For stable plant expression, a Hind III/Eco RI fragment was cloned into pBG(dAbs)BIN (Fig. 1A). For transient plant expression, a Hind III/Xho I fragment was filled in by the Polymerase fragment unit (Pfu, Stratagene), and blunt end-ligated into Sal I-linearized pP2C2S (Fig. 1B). For *in vitro *expression in transcription-translation systems, ScFv800E6 sequences from pEMBL-ScFv800E6 were PCR-amplified and subcloned into pIVEX 2.1 and pIVEX 2.2 vectors (Roche Applied Science), designed for the introduction of Strep II (Strep) tags at either the N-terminus or C-terminus (Fig. 1C and 1D). Fragments from pIVEX 2.1 and pIVEX 2.2 were excised and cloned into pIVEX 2.3d and pIVEX 2.4d to express polyhistidine (6XHis)-tagged ScFvs, (Fig. 1F and 1G). The two clones with C-terminal tags (Fig. 1C and 1E) were then linearized with Xho I, blunted, and re-circularized by ligation to bring the tag in frame with the open reading frames. A construct with a 27 residue-long spacer arm between the N-terminal His-tag and the coding sequence was produced by transferring the insert from pEMBL-ScFv800E6 into the polylinker of pIVEX 2.4d using Not I-Hind III adapters (Fig. 1G). The resulting ScFvs are shown (Fig. 1H). A control ScFv with irrelevant specificity (*to Citrus Tristeza Virus*, ScFvαCTV) was expressed in bacteria [22].

### Stable expression of ScFv800E6 in plants

Plant biology protocols were carried out as described [5], according to standard procedures [23]. Stable expression of ScFv800E6 in plants has been described [13]. Briefly, bacterial cultures of the *A. tumefaciens *GV3101 strain harboring pBG(dAbs)BIN-ScF800E6 were used to transform leaf disks from *Nicotiana tabacum*, and transgenic leaf disks selected in the presence of kanamycin. One shoot per leaf disk was grown *in vitro *in a climatic chamber, and plant RNA was analyzed by RT-PCR for the expression of VH sequences. Positive transgenic plants and their progeny were grown in a containment greenhouse, leaf tissues were homogenized [4], and total proteins were analyzed by Western blot.

### Transient expression of ScFv800E6 in plants

*Nicotiana benthamiana *plants were grown up to the 6 leaves state in a controlled greenhouse. *In vitro *transcripts generated from 1 μg of Spe I-linearized pP2C2S-ScFv800E6 were used for infection by rubbing leaves dusted with celite [24]. Tissues were collected seven days later, frozen in liquid nitrogen, and proteins were extracted in 0.05 M Tris-HCl pH 8.0/0.3 M NaCl/0.01 M PMSF/0.005 M ascorbic acid (5 ml per g of frozen tissue), homogenized, sonicated at 100 Watts (3 times, 10" each), ultra-filtered and concentrated (20×).

### Transcription-translation of ScFv800E6 *in vitro*

The different pIVEX-ScFv800E6 proteins (Fig. 1H) were expressed using the RTS 100 *E. coli*, a newly developed *E. coli *cell-free expression system for disulfide bonded proteins, according to the manufacturer's protocol (Roche Applied Science), in a ProteoMaster instrument [25]. This is based on the development, extensively described by Kim and Swartz [18], of a transcription-translation system involving several major novelties: (a) the inactivation of disulfide-reducing activities contained in a standard *E. coli *S30 extract, (b) the use of a glutathione redox buffer, (c) pH optimization, (d) the addition of GroE chaperones, and (e) a semi-continuous exchange dialysis format to achieve longer expression reactions. A conventional, reducing cell-free expression system was also used in control experiments. The UK construct and the *E. coli *chaperone DnaK are also from Roche. Brij 35 is from Calbiochem/EMD Biosciences, San Diego, CA. His-tagged ScFvs were purified by metal-chelate affinity chromatography on Ni-NTA agarose columns (Qiagen).

### ScFv testing

All ScFv preparations were tested for their ability to bind ErbB-2^+ ^cells by flow cytometry. The binding of ScFvs and mAbs was revealed using fluorescein isothiocyanate (FITC)-labeled rabbit antibodies to whole murine Ig (Cappel/ICN, Aurora, OH) at 50 μg/ml in the second step, unless noted otherwise. Where noted, antimurine Ig from a different vendor (Dako, Glostrup, Denmark) was also used. Phycoerythrin (PE)-conjugated Strep-Tactin was from IBA (Goettingen, Germany). An ELISA binding assay was carried out as follows: 96 well PVC microtiter plates (Corning, Acton, MA) were coated overnight with purified mAb 800E6, Fab' 800E6 and ScFv800E6 (approximately equimolar concentrations of 6.0, 2.0 and 1.0 μg/ml, respectively, in 0.1 M NaHCO_3 _pH 9.5). Following 3 washes with NaCl-tween (saline containing 0.05% Tween 20), adsorbed mAbs and fragments were incubated for 1 h with FITC-labeled rabbit antibodies to whole murine Ig (50 μg/ml, e.g. the concentration used in immunofluorescence). After washing with NaCl-tween, binding of the FITC-labelled antibody was revealed by 1 h incubation with peroxidase-conjugated goat anti rabbit Ig (Cappel/ICN), followed by washing and color development using O-phenylenediamine (Sigma-Aldrich) as substrate. Cells were metabolically labeled by incubation for 18 h in ^35^[S]-methionine (3.7 MBq/ml) containing medium, solubilized by the nonionic detergent NP40, and immunoprecipitated with protein A-sepharose 4B immunoadsorbents (Amersham) pre-loaded with rabbit antimurine Ig and either ScFvs or mAbs. Equilibrium binding studies were performed by incubating affinity purified antibodies and recombinant ScFvs (radiolabeled to a specific activity of 185 MBq/mg with ^125^I by the Chloramine T method) with target cells (1 × 10^5 ^per well in 100 μl volumes) in membrane-sealed 96 well plates (Millipore, Billerica, Ma) allowing instantaneous removal of free ligands by vacuum manifold filtration. Values of bound and free ligands were plotted according to the linear transform of the Law of Mass equilibrium, and the best fit of experimental data determined by regression analysis. All these procedures and Scatchard plot analysis are described in detail elsewhere [20].

### Immunohistochemistry

Human breast carcinoma specimens were obtained in the course of ablative surgery, in compliance with informed consent procedures. Four μm frozen sections, fixed in cold acetone for 10 min., were immunostained using a biotinylated anti Ig secondary antibody, and a streptavidin-biotin detection kit (Super Sensitive Detection Kit, Menarini, Florence, Italy), and the samples were counterstained with Mayer hematoxylin.

## Results

### Specificity of ScFv800E6

**Figure 2:**
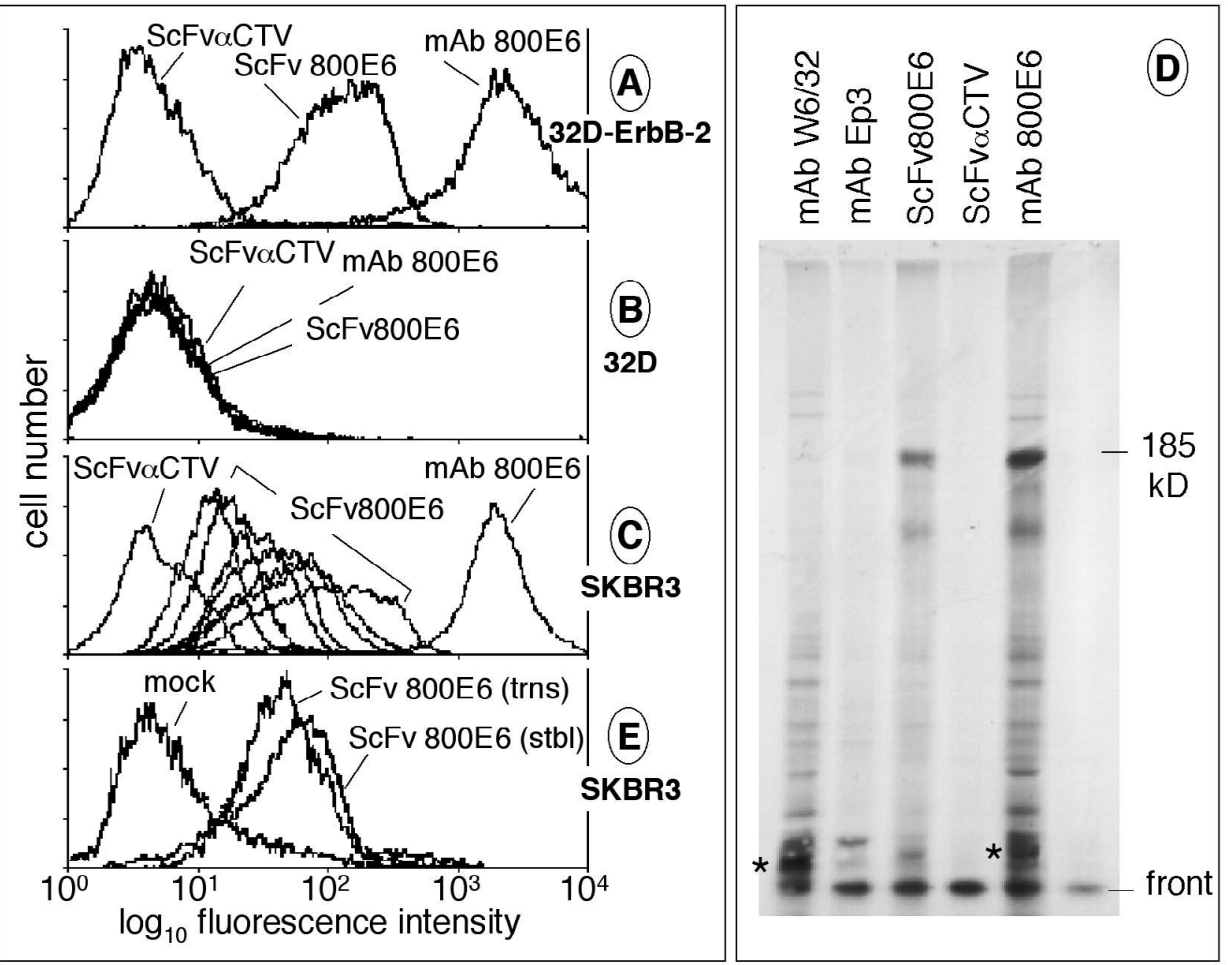
**Flow cytometry and immunoprecipitation with ScFv 800E6 produced in *E. coli *and transgenic plants**. Lysates (50 μl) from bacteria expressing either ScFv800E6 or ScFvαCTV, and the parental mAb 800E6 were incubated with 32D-ErbB-2 transfectants and ErbB-2-negative 32D cells (panels A and B, respectively), and revealed by an FITC-labeled antibody to whole murine Ig. Panel C: two-fold dilutions of bacterial lysates containing ScFv800E6 were tested as above on ErbB-2^+ ^SK-BR-3 cells. ScFvαCTV and mAb 800E6 are reported for comparison. Panel D: metabolically radiolabeled SK-BR-3 cells were lysed and immunoprecipitated with the indicated antibodies and ScFvs. Asterisks mark a group of closely migrating background bands of unknown origin present in several lanes. These are unlikely to represent MHC class I heavy chains that are poorly, if at all, expressed by SK-BR-3 cells [30]. Panel E: Lysates (50 μl) from tobacco plantulas stably (stbl) or transiently (trns) expressing ScFv800E6, and from transiently mock-infected plants (as a representative negative control) were incubated with SK-BR-3 cells, and ScFv binding revealed by flow cytometry as above.

Preliminary experiments were performed using crude bacterial lysates to compare the binding of ScFv800E6 and its parental antibody, mAb 800E6. Both reagents bound ErbB-2 transfectants but not parental ErbB-2-negative cells, as expected, although the former was 7–10 times weaker than the latter. An irrelevant ScFv to *Citrus Tristeza Virus *(ScFvαCTV) did not stain either cell line (Fig. 2A and 2B). In spite of the different binding intensities, the two reagents concordantly estimated ErbB-2 surface expression in a panel of breast carcinoma cell lines known [21] to express a wide range of ErbB-2 levels (Table 1). ScFv800E6 was titratable upon serial dilution (Fig. 2C), further supporting its binding specificity. In addition, ScFv800E6 and mAb 800E6 immunoprecipitated an identical 185 kD band from soluble extracts of metabolically radiolabeled SK-BR-3 cells (Fig. 2D).

**Table 1:** Reactivity of ScFv 800E6 with ErbB-2 transfectants and human cell lines

**Cell line**	**Lineage**	**CTVαScFv**	**ScFv800E6**	**mAb 800E6**	**source/ref**
32D	Myelomonocyte Leukemia (murine)	7^1^	7	7	[21]
32D-ErbB-2	Myelomonocyte Leukemia (transfectants)	6	171	2430	
SK-BR-3		4	278	2256	ATCC/[21]
MDA-MB-361		3	60	399	
ZR-75-1	Breast Ca	2	47	289	
DAL1		8	44	155	our lab
MCF7		3	19	76	ATCC/[21]
T47D		2	18	50	
End 9	Endometrial Ca	2	2	21	our lab
CRL1	Melanoma	6	7	10	
BE	Melanoma	1	2	20	[21]
ES-1	Melanoma	0	2	22	
A431	Epidermoid Ca	0	0	10	
SKOV3	Ovary Ca	1	1	10	

^1^Mean fluorescence intensity (m.f.i.) values estimated by flow cytometry after subtraction of background staining in the presence of PBS + anti murine Ig-FITC (background m.f.i. ranged from 5 in 32D cells to 15 in the other cell lines). Cut-off levels can be arbitrarily set at values twice the highest background, e.g. above 15.

### Stable and transient expression of ScFv800/E6 in tobacco plants

Upon stable plant transformation, RT-PCR of putative transgenics revealed that 56% of them expressed ScFv800E6 transcripts. Extracts from selected positive plants were analyzed by Western blotting and estimated to contain ScFv polypeptides at a concentration of 0.6–0.8 μg/g of plant tissue (not shown). Similarly, ScFv800E6 was detected by Western blot in extracts from both directly and systemically infected leaves of transiently modified *Nicotiana benthamiana *plants. Much higher yields (800 μg/g of plant tissue) were obtained from leaves exhibiting systemic symptoms (data not shown). Extracts (50 μl) containing 0.14 μg/ml and 160 μg/ml of ScFv800E6 from stable and transient transgenic plants, respectively, were used to stain SK-BR-3 cells in indirect immunofluorescence. A representative flow cytometry analysis at the widely different antibody concentrations noted above revealed that the two preparations similarly bound SK-BR-3 cells (Fig. 2E). Binding intensities were comparable to (or slightly lower than) the intensities seen with bacterial extracts on the same target cells (compare panels C and E), indicating that saturating concentrations of ScFv had been reached. Quite puzzling, ScFv binding could not be detected using an antibody to a c-Myc tag present on the ScFvs produced in bacteria and plants (not shown), hampering purification. Based on these findings, before embarking on the production of additional transgenic plants, we resorted to *in vitro *transcription-translation systems to rapidly obtain a set of modified ScFv800E6 proteins to be used for the identification of optimal folding conditions and for the evaluation of tagging and purification strategies.

### Expression of ScFv800E6 in cell-free *in vitro *transcription-translation systems

**Figure 3:**
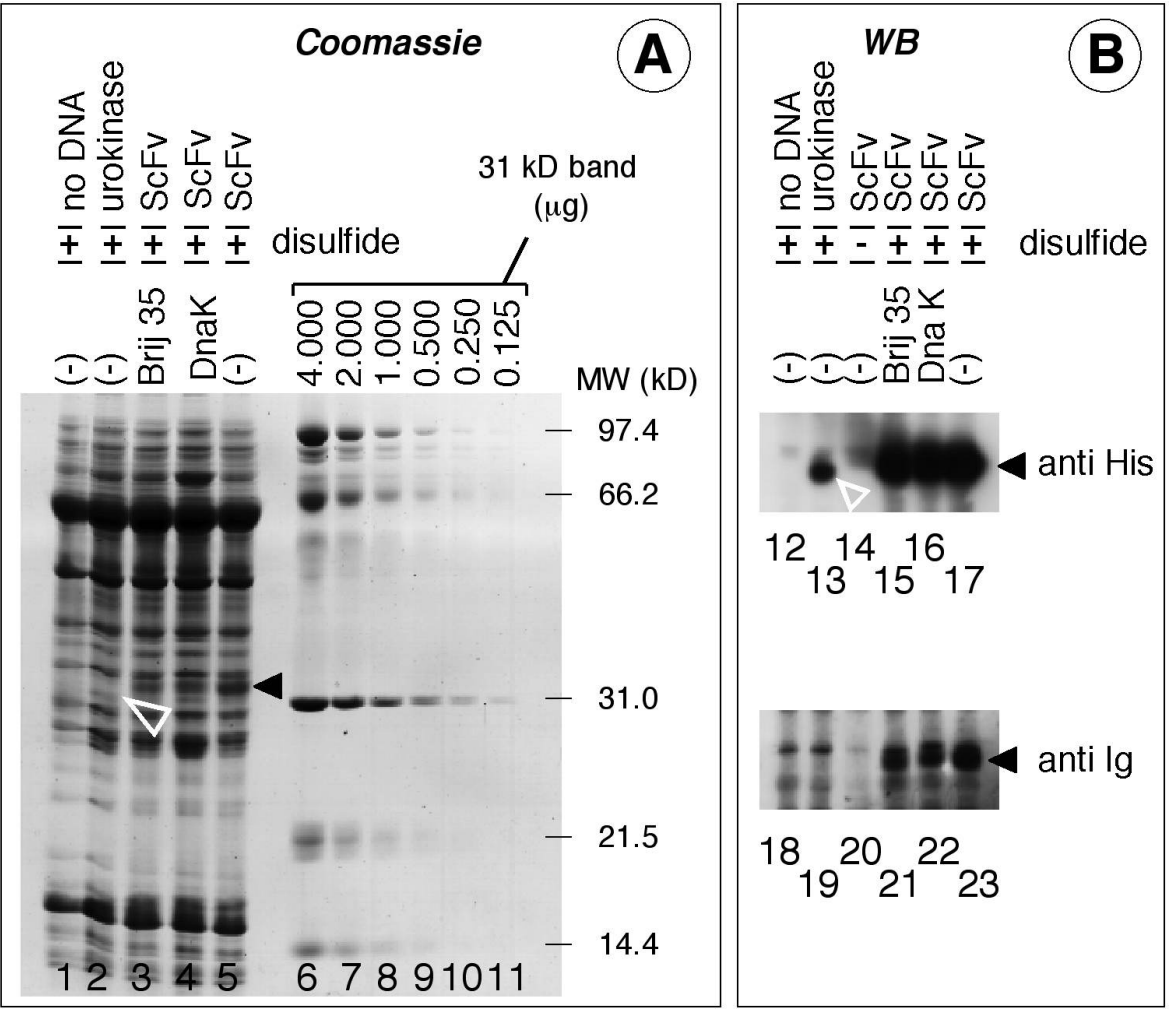
**Biochemical analysis of tagged ScFv 800E6 produced in a cell-free transcription-translation system**. Panel A: His(6x)-ad-N-ScFv800E6 and urokinase (UK) were transcribed-translated either in a conventional mix (disulfide -), or in a mix promoting disulfide bonding (disulfide +), as indicated, with and without Brij 35, or the chaperone DnaK. Transcription-translation was also carried out in parallel in the absence of template DNA. Supernatants (5 μl) of transcription-translation mixtures were run on a SDS-PAGE slab, side by side with two-fold dilutions of MW standards containing known amounts of a 31 kD protein, and the gel was stained by Coomassie blue. Panel B: Smaller volumes (1 μl) of the same supernatants were run as above, and electroblotted. The filter was stained with anti His-tag and anti Ig antibodies. Open and closed arrowheads mark UK and ScFv800E6 polypeptides.

ScFv800E6 constructs were prepared in 4 distinct pIVEX vectors, each of which Strep- or His-tagged at the N- or C-terminus (Fig. 1C–F), plus a fifth construct with a N-terminal His-tag on the tip of a 27 residue-long spacer arm (Fig. 1G). The Strep tag was selected because it is particularly useful for flow cytometry detection, since it is recognized with particularly high affinity by fluorescent, recombinant Strep-Tactin. Because the ScFvs contain two disulfide bonds, the five constructs were expressed not only in a conventional *E. coli*-based cell-free transcription-translation system, but also in a newly developed transcription-translation system for disulfide-bonded proteins. The control urokinase (UK) protein, that contains 6 disulfide bonds, was transcribed-translated in parallel. Supernatants from representative His(6x)-ad-N-ScFv800E6 transcription-translation mixtures were run on a SDS-PAGE gel, and either stained by Coomassie blue (Fig. 3A) or Western blotted by peroxidase-conjugated antibodies to the His-tag and murine Ig (Fig. 3B). His(6x)-ad-N-ScFv800E6 (solid black arrowheads: lanes 3, 4, 5, 15, 16 and 17) and UK (open white arrowheads: lanes 2 and 13) displayed the expected electrophoretic mobilities (approximately 32 kD in both cases), and were absent in lanes loaded with mock-transcribed-translated mixes (lanes 1, 12, and 18). Neither the nonionic detergent Brij 35 (1%) nor the *E. coli *chaperone DnaK significantly increased ScFv yield (compare lanes 3 and 4 to 5; 15 and 16 to 17; 21 and 22 to 23). In contrast, His(6x)-ad-N-ScFv800E6 was barely detectable when translated in a conventional, reducing system (lanes 14 and 20). Under these conditions the ScFv could be mainly recovered in the insoluble fractions (data not shown).

In comparison with the 31 kD molecular weight marker in lane 8, the amounts of the ScFv component synthesized in a disulfide-capable format (lane 5) could be estimated to be approximately 1 μg of protein, which corresponds to a concentration in reaction mixtures of 200 μg/ml, 10-fold greater than that of most mAbs in hybridoma supernatants. The yield of the ScFvs was approximately 2.5% of the total proteins contained in the mix at the end of the transcription-translation process. All the ScFvs remained soluble even after repeated (up to 3 times) freeze-thawing cycles with no appreciable loss in reactivity (not shown). Thus, considerable yields and enrichment can be obtained in transcription-translation systems, as long as proper disulfide bonding of ScFv is ensured *in vitro*.

### Flow cytometry with ScFvs produced in cell-free transcription-translation systems

**Figure 4:**
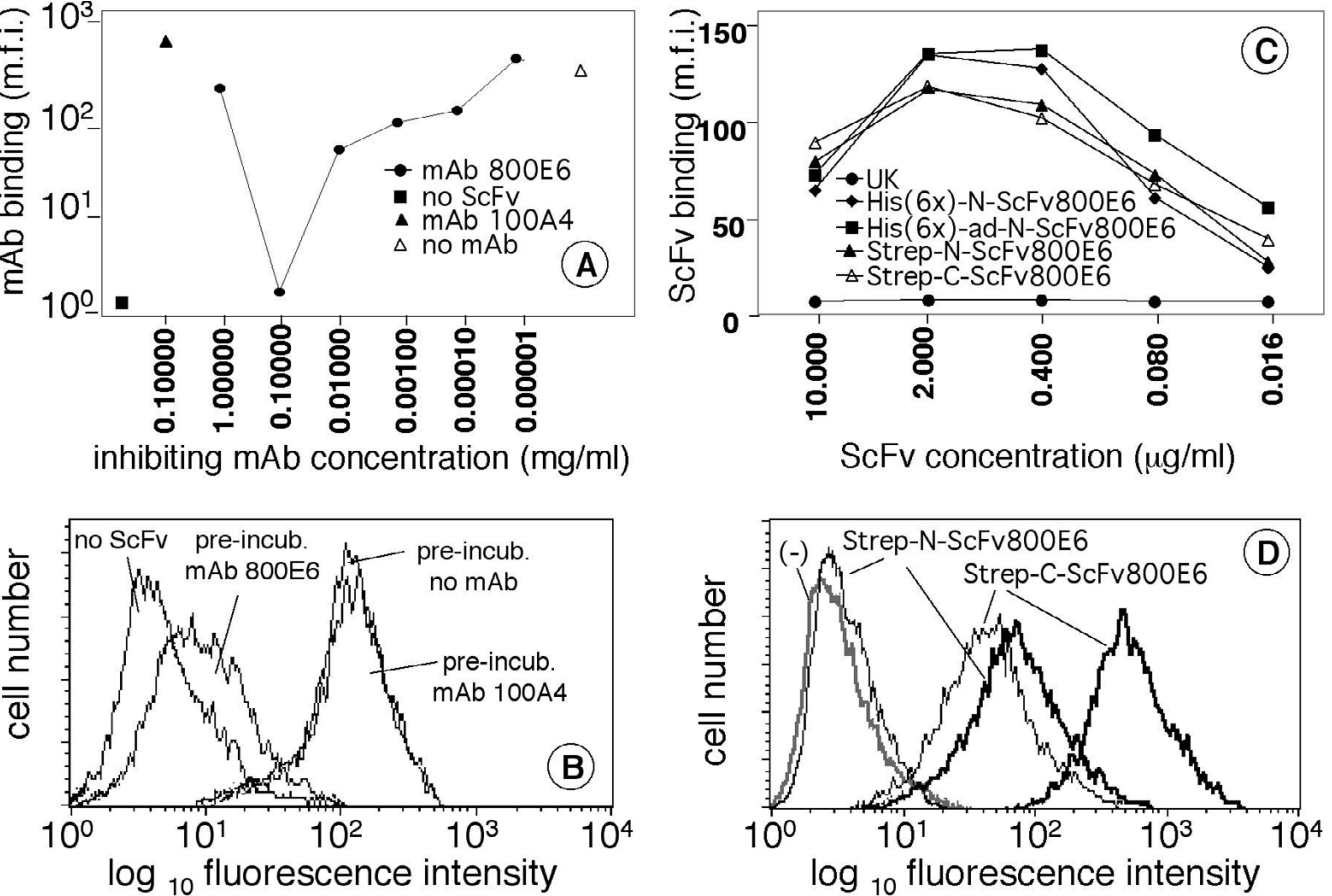
**Comparative flow cytometry analysis of different ScFvs to ErbB-2 and epitope blocking**. Panel A: mAb 100A4 (0.1 mg/ml) and mAb 800E6 (at the different, indicated concentrations) were incubated for 30 min with SK-BR-3 cells. His(6x)-ad-N-ScFv800E6 from transcription-translation mixtures (at a final concentration of 1 μg/ml) was then added and tested for its ability to bind SK-BR-3 cells using a rabbit antibody to the His-tag followed by an FITC-labeled antibody to rabbit Ig. Binding of the His-tagged ScFv in the absence of competing antibody (no mAb), and background staining in the absence of ScFv (but in the presence of both anti His-tag and FITC-labeled antibodies; no ScFv) are also shown. Mean fluorescence intensities (m.f.i.). Four selected experimental points of the experiment in panel A (including maximal inhibition by mAb 800E6 at 0.1 mg/ml) are shown in panel B. Panel C: five-fold dilutions of the indicated ScFv and UK preparations were tested by flow cytometry for their ability to bind SK-BR-3 cells, and revealed by FITC-labeled anti Ig antibodies. Panel D: Strep-N-ScFv800E6, Strep-C-ScFv800E6, and a mock transcription-translation mixture (-) were tested in flow cytometry for their ability to bind SK-BR-3 cells using either PE-Strep-Tactin or PE-streptavidin (thick and thin lines, respectively).

To assess the activity and fine specificity of the ScFvs produced in the transcription-translation system, we tested the ability of the parental mAbs and His(6x)-ad-N-ScFv800E6 to inhibit each other in flow cytometry. Pre-incubation of SK-BR-3 cells with a wide range of mAb 800E6 concentrations, but not mAb 100A4 to a distinct ErbB-2 epitope [19], proportionally (and up to 95%) inhibited the binding of His(6x)-ad-N-ScFv800E6 (Fig. 4A), with a clear prozone effect (a paradoxical reduction of inhibition at high mAb concentrations) at 1 mg/ml. Four selected points are also analytically displayed in panel B. Lesser, but still significant, levels of competition for the 800E6 epitope were seen when ScFv800E6 was used in the pre-incubation step (not shown). Thus, the binding of the disulfide-bridged ScFv can be competed by, and retains the fine epitope specificity of, the parental antibody.

All the 5 tagged pIVEX-ScFvs were transcribed-translated in disulfide mixtures, and tested by flow cytometry at 5 different dilutions using the same FITC-labeled secondary antibody to murine Ig. ErbB-2 was detected on SK-BR-3 cells with comparable efficiency and with a similar prozone effect (Fig. 4C). The binding of all the ScFvs displayed the highest mean florescence intensity (m.f.i.) value (140 approximately) at similar concentrations (in the microgram per ml range). This concentration is equivalent to 20 ng per 5 × 10^5 ^target cells. Two pIVEX-ScFvs bearing a suitable tag (Strep-N-ScFv800E6 and Strep-C-ScFv800E6) were also tested in flow cytometry using PE-conjugated Strep-Tactin and PE-conjugated streptavidin as secondary reagents (Fig. 4D). Four observations were made: (a) the C-terminally tagged ScFv performed much better than the N-terminally tagged one; (b) the fluorescence intensity conveyed by Strep-Tactin was in each case much stronger than that conveyed by conventional streptavidin, to the extent that the weak binding of Strep-N-ScFv800E6 was essentially undetectable by the latter; (c) the optimal combination (Strep-C-ScFv800E6 followed by Strep-Tactin) resulted in a substantial improvement in m.f.i. values as compared to the same ScFv followed by antimurine Ig secondary reagents conjugated with either FITC or PE (m.f.i of 700 vs. 140; compare with Fig. 4C); (d) these m.f.i. values were closer to those typical of the parental mAb 800E6 followed by conventional secondary antimurine Ig reagents conjugated with either FITC or PE (m.f.i. values > 1300 in all the experiments that were performed; see Fig. 2 and data not shown).

Similar to the strep II tag, a preference for C-terminal tagging was also seen with His-tagged ScFvs and anti His secondary antibody, but the introduction of a spacer arm, as in His(6x)-ad-N-ScFv800E6, restored the availability of the N-terminal epitope tag (not shown). It can be concluded that: (a) all the variants of ScFv800E6 can be produced in a cell-free system at concentrations and amounts similar to those recommended for flow cytometry and immunohistochemistry with most monoclonal antibodies, (b) the type and position of the tags have no detectable effect on ScFv binding (i.e. tags do not interfere with the antigen binding site), whereas the tag position is important in order to make the tag available for secondary reagents, and (c) Strep-Tactin outperforms streptavidin. Thus, optimization in the position and type of tag, as well as selection of appropriate secondary reagents, largely compensate for the reduced performance of ScFvs in comparison to their parental mAb.

### Conservation in the antigen binding site of ScFvs

We then addressed the issue of the reduced performance of ScFvs in comparison to their parental mAb when evaluated by conventional secondary anti murine Ig reagents. This may reflect one or more of the following: (a) a reduced recognition by secondary antibodies due to the removal of Fc epitopes from ScFv800E6; (b) a reduced binding of the ScFv due to monovalency; (c) a reduced binding performance of the ScFv due, among other possible causes, to any of the following: (i) alterations in crucial amino acid sequences consequent to cloning antibody sequences into an ScFv format; (ii). improper folding of the antigen binding site upon *in vitro *translation of mammalian sequences in prokaryotic cell-free systems; (iii) ScFv dimerization.

To directly address (a), we tested the FITC-labeled antibody to whole mouse Ig used in flow cytometry for its ability to bind mAb 800E6, its monovalent Fab' fragment (as a control), and affinity-purified His(6x)-ad-N-ScFv800E6, taking advantage of the ELISA assay described in methods. The binding of FITC-labeled secondary antibodies (from two different commercial sources, see methods) to mAb, Fab and ScFv adsorbed on PVC plates in equimolar amounts displayed the following ratios: 10:5:1.

**Figure 5:**
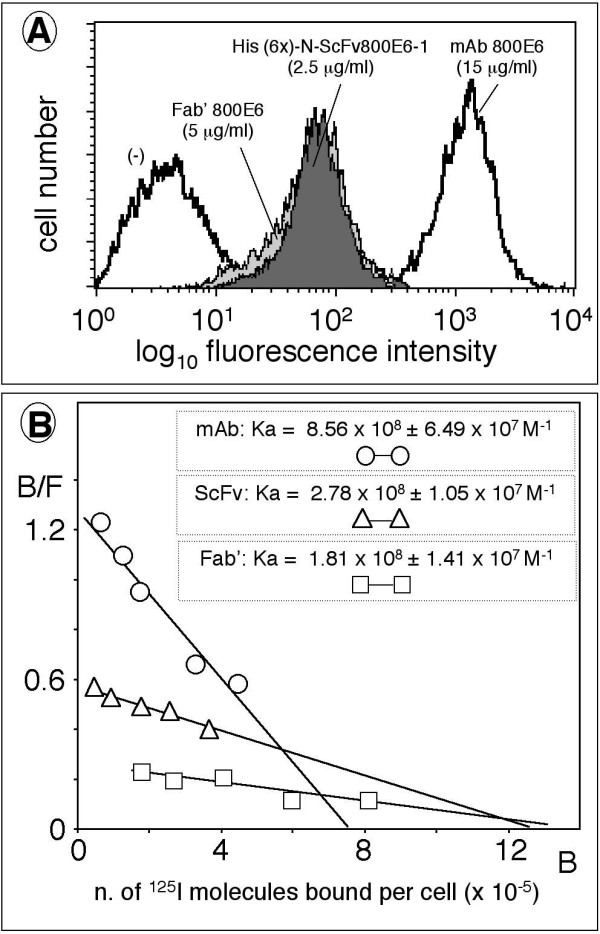
**Flow cytometry and Scatchard plot analysis with ScFv800E6 produced in transcription-translation systems and tobacco leaves**. Panel A: Equimolar amounts of His(6x)-ad-N-ScFv800E6, Fab' 800E6 and parental mAb 800E6 were compared by flow cytometry for their ability to bind SK-BR-3, using a conventional FITC-labeled secondary antibody to whole murine Ig. Panel B: the binding of ^125^I-labeled mAb 800E6 and His(6x)-ad-N-ScFv800E6 to SK-BR-3 cells at equilibrium was expressed in the form of bound (B) ligand versus the ratio of bound over free (B/F) ligands. The slope of the best-fit curve of individual determinations is directly proportional to the observed binding affinity (Ka: association binding constant). The intercept on the abscissa is an extrapolation to infinity of the number of ErbB-2 epitopes per cell multiplied by the valence of the ligand.

These results are consistent with the removal of some and most framework Ig epitopes from the Fab' and ScFv, respectively, and quantitatively agree with the 7–10 fold difference in binding intensities between the ScFv and the parental mAb, routinely detected in the above flow cytometry studies. Having estimated their reactivity with FITC-labeled secondary antibodies, the three reagents were again compared, in the same flow cytometry experiment, for their ability to bind SK-BR-3 cells. Optimal working dilutions of the three reagents were determined as shown in the experiments displayed in figures 2C and 4C to rule out the possibility of prozone effects, and staining was performed at the indicated, selected equimolar concentrations. Representative results with mAb, one of two preparations of Fab', and ScFv, followed by one of the two FITC-labeled secondary antibodies that were employed (Fig. 5A) revealed an approximately 10:1:1 ratio in the binding of the three reagents, with the Fab and the ScFv displaying extremely similar binding intensities. The ten-fold drop in the ScFv performance was confirmed, and correlated with the ten-fold reduced recognition of ScFv vs. mAb detected by the secondary antibody in the ELISA binding assay. In contrast, the ten-fold drop in Fab' performance exceeded the two-fold reduced recognition by the secondary antibody.

These results altogether provide further support for (a), and suggest that (b), e.g. reduced binding due to monovalent interactions, is also involved, although a more direct method is needed to account for the different extent of this reduction in the case of monovalent Fab' and ScFv. Then, we directly measured the equilibrium binding affinities of ^125^I-labeled mAb800E6, Fab' 800E6, and His(6x)-ad-N-ScFv800E6, and determined the valence of the parental mAb. Equilibrium binding studies demonstrated a high binding affinity (Ka in the 10^8 ^M^-1 ^range) for all the three ligands (Fig. 5B), and detected the expected drops in affinity of the ScFv and Fab' as compared to the parental antibody. These were estimated to be approximately 3-fold and 4.5-fold, respectively. A double number of ScFv and Fab' as compared to mAb binding sites (intercept on the abscissa) was consistent with mAb 800E6 being the only reagents capable of bivalent interactions. Altogether, the limited range of variation in the observed association binding constants, and the detection of monovalent vs. divalent binding demonstrated that the absence of Fc epitopes (a) and monovalent binding (b) largely explain the apparent low staining efficiency of ScFv800E6 when using conventional secondary anti Ig reagents, whereas alterations and/or mis-folding (c) of the ScFv play a minor, if any, role in reducing its binding performance. Finally, a comparison of Fab' and ScFv binding abilities in different assays indicates that enzymatic fragmentation of the natural antibody is more deleterious in our hands than cloning and expression in a recombinant form of ScFv800E6 sequences.

### Use of ScFv800E6 for the immunohistochemical staining of breast cancer lesions

**Figure 6:**
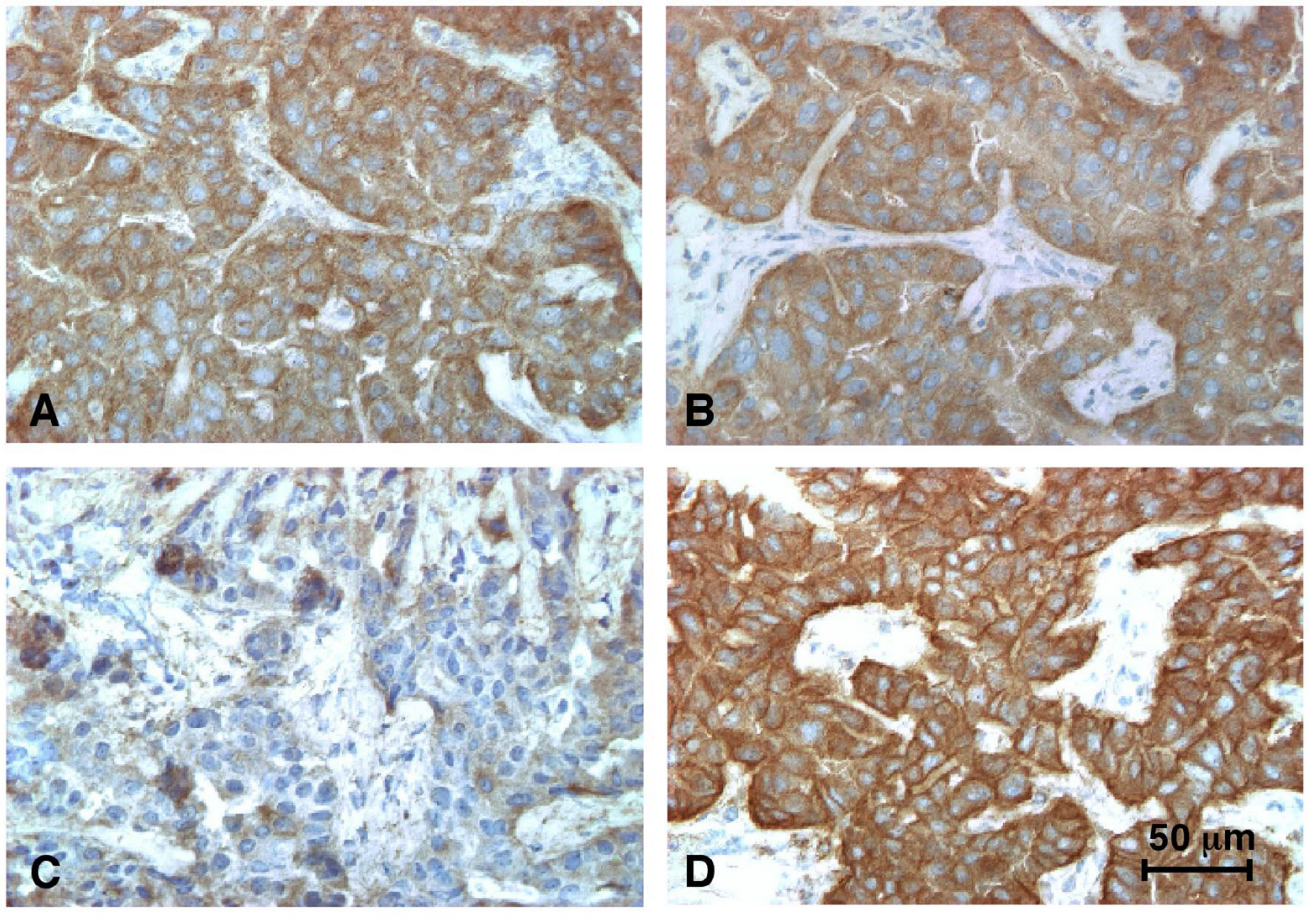
**Immunohistochemistry of breast carcinoma lesions with different preparations of ScFv800E6**. Semi-serial cryostatic sections of a primary ductal breast carcinoma lesion were stained with (A) 50 μl of His(6x)-N-ScFv800E6 transcription-translation mixes; (B) 50 μl of extracts from plants transiently expressing ScFv800E6; (C) 50 μl of extracts from wild-type plants; and (D) 50 μl of mAb 800E6 (50 μg/ml) in PBS/5% FCS. ScFv/mAb binding was revealed as described in Materials and Methods.

To provide evidence that recombinant ScFvs and phytoantibodies may be useful in a diagnostic setting, semi-serial cryostat sections of breast carcinoma lesions were stained with different ScFv800E6 preparations. Representative results (Fig. 6) demonstrate that all of them clearly and reproducibly discriminated ErbB-2^+ ^from ErbB-2^- ^breast carcinoma lesions.

## Discussion

In this report we have exploited promising, alternative expression systems to produce and test ScFv800E6 as a candidate molecule for applications in oncology. ScFv800E6 is the first ScFv to ErbB-2 produced in plants. Along with its 5 tagged variants, it is also one of the few ScFvs to be developed for expression in second-generation, high-yield, cell-free transcription-translation systems capable of establishing disulfide link formation.

We have shown that the binding of ScFv800E6 obtained from different platforms is antigen-specific, saturable, titratable, and can be competed by the parental antibody, i.e. it recapitulates the canonical features and the fine specificity of a natural ligand-target interaction. Radiobinding studies and flow cytometry data were consistent with a remarkably robust, stable, versatile, and modular ScFv800E6 'backbone' that tolerates extensive modifications at both the N- and C-termini, although the position of the tag is crucial for its availability upon incubation with secondary reagents. The apparently low staining efficiency of ScFv800E6 was largely due to the use of conventional secondary anti-Ig reagents and not to a low binding affinity, since ScFv binding, in flow cytometry, was at least as high as that of the monovalent Fab' in spite of the use of secondary reagent that preferentially bound to the latter. Accordingly, equilibrium binding studies revealed a binding affinity slightly higher than that of the Fab' with no major drop as compared to that of the parental, bivalent antibody. This is remarkable, since bivalent binding is known to greatly stabilize antigen-antibody complexes [26]. These results suggest that the antigen binding site of the recombinant ScFv has undergone no major derangements as compared to that of the natural antibody, whereas enzymatic fragmentation may moderately hamper the performance of the Fab'. Thus, expression of recombinant ScFv800E6 bypasses a potential obstacle that would preclude size reduction of the parental 800E6 antibody.

ScFv800E6 can be produced in all the expression platforms at concentrations (see below) sufficient, or greater than required, for all the major indirect trace binding assays, and all the ScFv variants perform satisfactorily with no need to modify or adapt commercially available immunodiagnostic reagents and kits (e.g. in immunohistochemistry). ScFvs can be tagged for detection by an extremely sensitive secondary reagent, such as Strep-Tactin, that outperforms even sensitive streptavidin-based detection systems and largely compensates for monovalent binding. ScFvs can be radiolabeled to high specific activity for *in vivo *radioimaging by a standard Chloramine T iodination, with no need for special procedures or dedicated protocols. In summary, ScFv800E6 variants are all ready for application in oncology. In this respect, two issues are of particular interest: yield and folding.

We observed that the yield of ScFv800E6 from stable transgenic plants did not exceed the microgram per ml range, i.e. it was low as compared to other ScFvs produced in tobacco plants [reviewed in [6]]. Strikingly, an improvement of three orders of magnitude was obtained by recovering the ScFv800E6 from leaves exhibiting systemic symptoms in transiently modified plants, indicating that the features of ScFv800E6 are not intrinsically incompatible with its efficient expression in plants. Because preliminary data indicate that transgene silencing may affect ScFv expression in stable transgenic plants, we are currently improving ScFv yield by taking advantage of plant expression systems that alleviate this problem and in addition dispatch antibody fragments to specific plant compartments such as roots and seeds.

As an alternative to plant expression, we produced ScF800E6 in a cell-free transcription-translation system that, as described in methods, differs under several respects from other cell-free systems previously used to express disulfide-bonded ScFvs [6,8]. ScFv800E6 was obtained with a yield of 200 μg/ml, approximately 20 times higher than that of a different ScFv produced in a conventional format [6]. A systematic comparison involving transcription-translation of many different ScFvs in the available formats is mandatory to determine whether optimal conditions must be worked out individually for every construct or, alternatively, similar protocols can be used for different ScFvs.

Cell-free expression of ScFvs is also relevant in the context of the 'ribosome display' approach. This approach takes advantage of transcription-translation for the phenotypic selection of ScFvs with increased affinity upon their immobilization on polysomes [27,28]. In principle, it should be possible to incorporate the present semi-continuous format in current ribosome display protocols, offering a high-yield alternative to experiments aimed at ScFv improvement.

A surprising finding becomes apparent when the results in the various expression platforms are compared. ScFv800E6 was functional when expressed in reducing cytosolic environments (bacteria and plants), but not in transcription-translation systems unable to establish disulfide linkage (Figs. 3 and 4). At least two interpretations may be proposed: (a) some or all of the ScFv molecules synthesized *in vivo *are functional because they somehow manage to get disulfide-linked in the cytosol or other cellular locations; (b) disulfide linkages are essential *in vitro *but not *in vivo*, resulting in two different (but similarly active) ScFv folds. These issues are of bio-technological relevance in view of large-scale production. However, to be addressed they require structural studies on large amounts of purified ScFvs from different sources. Whatever the exact folding mechanism, and the role of disulfide linkage, ScFv800E6 is stable and active in different expression platforms. This property is highly desirable and unusual among previously described recombinant antibody fragments [29]. This results in unique versatility and flexibility in the choice of expression platforms. Therefore, ScFv800E6 appears to be an ideal candidate for a three-step development of bio-technological processes leading to progressive improvement of the reagent. As outlined in this report, reagents of this kind may be pre-screened for activity in different hosts, modified and tested on a small-medium scale in a convenient platform (e.g. cell-free systems), and then moved (if required) to another one (e.g. plants) for mass production, and the optimization loop may be repeated as many times as needed.

Plant and cell-free expression systems are largely complementary. The advantages of mass production of recombinant antibody fragments in plants have been long known [3]. Now, with the production of functional ScFvs *in vitro*, transcription-translation systems may also become extremely attractive. In this respect, it may be noted that although the available cell-free systems are designed to produce amounts of recombinant proteins in the milligram range, yield and final ScFv concentrations in our experimental system exceed those contained in hybridoma supernatants (Fig. 3). Moreover, scale-up is not a concern, since there is no theoretical upper limit to the reaction volume. This is in contrast to mammalian cell bioreactors that require sophisticated equipments in order to maintain adequate gas permeation and nutrient diffusion in the liquid phase, and at the same time prevent cell shearing.

## Conclusion

By cutting down cost and initial investments, ScFv production in alternative expression platforms may alleviate the foreseen deficit in manufacturing capacity (bio-manufacturing bottleneck) of mammalian cell bioreactors, and may become of special interest for a rapid translation of potentially interesting bio-molecules into the clinical use.

## Abbreviations

ScFv: Single chain Fragments of antibody variable regions; mAb: monoclonal Antibody; Ig: Immunoglobulin; FITC: fluorescein isothiocyanate; PE: Phycoerythrin; m.f.i.: mean fluorescence intensity; Ka: association (affinity) binding constant.

## Competing interests

FY was working at Roche Diagnostics when he made his experimental contribution to this work. ScFv800E6 is part of the European patent no. 94830567.7 filed on 7/12/1994.

## Authors' contributions

PGa, AL, IP, FN, MDN, MS, AT, and CC cloned Ig sequences and expressed them in bacteria and plants. FY prepared pIVEX constructs. FY and AM expressed them in transcription-translation systems. RF and PGN tested all the preparations by flow cytometry, and tested transcription-translation mixtures by SDS-PAGE and Western blotting. PGi performed equilibrium binding studies and Scatchard analysis. MB and MM carried out immunohistochemistry. All the authors participated in the preparation of the manuscript and approved the final version.
